# Integrating Patient-Reported Outcomes Into Prognostication in Gastroesophageal Cancer: Results of a Population-Based Retrospective Cohort Analysis

**DOI:** 10.1093/oncolo/oyae010

**Published:** 2024-03-02

**Authors:** Thais Baccili Cury Megid, Divya Sharma, Zeynep Baskurt, Lucy Xiaolu Ma, Xin Wang, Carly C Barron, Raymond Woo-Jun Jang, Eric Xueyu Chen, Carol Jane Swallow, Aruz Mesci, Jonathan Yeung, Rebecca K S Wong, Savtaj Singh Brar, Patrick Veit-Haibach, John Kim, Yvonne Bach, Hiroko Aoyama, Elena Elimova

**Affiliations:** Medical Oncology and Hematology, Princess Margaret Cancer Centre, Toronto, Canada; Biostatistics Division, University of Toronto,Toronto, Canada; Biostatistics Division, University of Toronto,Toronto, Canada; Medical Oncology and Hematology, Princess Margaret Cancer Centre, Toronto, Canada; Medical Oncology and Hematology, Princess Margaret Cancer Centre, Toronto, Canada; Medical Oncology and Hematology, Princess Margaret Cancer Centre, Toronto, Canada; Medical Oncology and Hematology, Princess Margaret Cancer Centre, Toronto, Canada; Medical Oncology and Hematology, Princess Margaret Cancer Centre, Toronto, Canada; Department of Surgical Oncology, Princess Margaret Cancer Centre,Toronto, Canada; Department of Surgery, Mount Sinai Hospital, Toronto, Canada; Department of Radiation Oncology, Princess Margaret Cancer Centre, Toronto, Canada; Division of Thoracic Oncology, Toronto General Hospital,Toronto, Canada; Department of Radiation Oncology, Princess Margaret Cancer Centre, Toronto, Canada; Department of Surgical Oncology, Princess Margaret Cancer Centre,Toronto, Canada; Department of Surgery, Mount Sinai Hospital, Toronto, Canada; Department of Medical Imaging, Princess Margaret Cancer Centre, Toronto, Canada; Department of Radiation Oncology, Princess Margaret Cancer Centre, Toronto, Canada; Department of Medical Oncology and Hematology, University of Toronto,Toronto, Canada; Department of Medical Oncology and Hematology, University of Toronto,Toronto, Canada; Medical Oncology and Hematology, Princess Margaret Cancer Centre, Toronto, Canada

**Keywords:** patient-reported outcomes, gastroesophageal cancer, prognostic, patient-centered care, ESAS, PRFS, ECOG

## Abstract

**Background:**

Patient-reported outcomes measures (PROM) are self-reflections of an individual’s physical functioning and emotional well-being. The Edmonton Symptom Assessment Scale (ESAS) is a simple and validated PRO tool of 10 common symptoms and a patient-reported functional status (PRFS) measure. The prognostic value of this tool is unknown in patients with gastroesophageal cancer (GEC). In this study, we examined the association between the ESAS score and overall survival (OS) in patients with GEC, the prognostication difference between ESAS and Eastern Cooperative Oncology Group (ECOG), and assessed the correlation between PRFS and the physician-reported ECOG performance status (PS).

**Methods:**

The study was a retrospective cohort study of 211 patients with GEC with localized (stages I-III) and metastatic disease who completed at least one baseline ESAS prior to treatment. Patients were grouped into 3 cohorts based on ESAS score. OS was assessed using the Kaplan-Meier method, and the concordance index (c-index) was calculated for ESAS and physician-reported ECOG. The agreement between PRFS and physician-ECOG was also assessed.

**Results:**

In total, 211 patients were included. The median age was 60.8 years; 90% of patients were ECOG PS 0-1; 38% of patients were stages I-III, while 62% were de novo metastatic patients. Median OS in low, moderate, high symptom burden (SB) patients’ cohorts was 19.17 m, 16.39 mm, and 12.68 m, respectively (*P* < .04). The ability to predict death was similar between physician-ECOG and ESAS (c-index 0.56 and 0.5753, respectively) and PRFS and physician-ECOG (c-index of 0.5615 and 0.5545, respectively). The PS agreement between patients and physicians was 50% with a weighted Kappa of 0.27 (95% CI: 0.17-0.38).

**Conclusion:**

Patient’s SB seems to carry a prognostic significance. ESAS and physician-reported ECOG exhibit comparable prognostic values. Physicians and patients can frequently have divergent opinions on PS. ESAS takes a patient-centered approach and should be encouraged in practice among patients with GEC as an additional tool for prognostication.

## Teaser

The prognostic value of the ESAS tool is unknown in gastroesophageal cancer. This study examined the association between the ESAS score and overall survival in patients with gastroesophageal cancer and assessed the correlation between patient-reported functional status and physician-reported ECOG performance status.

**Implications for Practice**

By using an established patient-reported outcome measure, this study is the pioneer in examining the link between the severity of symptoms and the probability of mortality in patients with gastroesophageal cancer (GEC). The report provides an analysis of patient-reported symptoms related to GEC, as well as evaluations against other traditional prognostic tools. Additionally, it is the first research study to contrast the performance status assigned by doctors versus that reported by patients concerning patients with GEC. The study results recommend the potential inclusion of alternative patient-centered tools in clinical practice.

## Introduction

There is a growing interest in integrating the collection of patient-reported outcomes (PROs) and their measures, referred to as PROMs, in routine clinical practice to enhance the quality of clinical care^1^. PROMs reflect how patients feel and function and are measured via questionnaires which assess symptoms experienced, quality of life, and performance status (PS) associated with the patient’s treatment and disease.^1^

Routine use of PROMs in clinical care has been shown to improve the patient-centered nature of care, resulting in increased symptom monitoring, improved quality of life, and decreased use of acute care services, such as emergency department visits.^2-4^ Additionally, integrating PROs into routine care has been associated with increased survival in randomized clinical trials that compared PRO monitoring in addition to standard imaging to standard scheduled imaging for detecting adverse event and symptomatic recurrence in patients with solid tumors in patients receiving treatment and in routine surveillance.^5,6^

The Edmonton Symptom Assessment Scale (ESAS) is a validated 10-item patient-rated symptom visual analog scale developed initially for use in assessing the symptoms of patients receiving palliative care and has been integrated into clinical workflow since 2007.^7^ Patients typically complete ESAS before their visits and rate the severity of symptoms, including pain, activity, nausea, depression, anxiety, drowsiness, lack of appetite, well-being, and shortness of breath, ranging from 0 to 10. Although developed for use in the palliative care setting, the ESAS has been validated in curative settings in multiple studies.^2,8^ In 2013, a version of the Patient-Reported Functional Status (PRFS) based on Eastern Cooperative Oncology Group (ECOG) PS was added to supplement the ESAS survey.^9^ As a result, the ESAS survey provides not only patient-centered well-being but also reflections on patients’ physical functioning.^10^ ESAS is offered for all patients with cancer during their visits for more than 15 years in Canada.

Gastric and esophageal cancers affect approximately 1 100 000 and 600 000 patients worldwide, respectively, and together represent around 9% of all neoplasia.^11,12^ Patients with advanced gastroesophageal carcinoma are generally offered palliative treatment and prognostication is crucial for patients, caretakers, and oncologists, allowing them to make informed decisions surrounding medical interventions, achieving preferred places of care, and engaging in advance care planning.^13^ ECOG has been used as the main tool for prognostication in the gastroesophageal population for several decades^14,15^; however, it is essential to acknowledge that ECOG does come with certain limitations. Its evaluation by physicians makes it subjective and susceptible to bias. Furthermore, its assessment does not encompass factors like symptom burden (SB), which are essential aspects of overall health evaluation.^16^

Patients with gastroesophageal cancer experience severe SB of disease including nausea, dysphagia, and pain.^17^ Several studies have shown that baseline SB not only evaluates the symptoms experienced by patients but also serves as a predictive tool for survival outcomes in individuals with advanced solid tumor disease.^18-20^ PRO and prognostication remain an understudied topic in the gastroesophageal population. This study aimed to (1) investigate the role of PRO in the care of patients with gastroesophageal adenocarcinoma by determining whether there is an association between baseline symptoms and OS, (2) compare the prognostic ability between ESAS and ECOG (evaluated by physicians), and (3) evaluate PS agreement rates between physicians and patients.

## Methods

### Study Population and Data Collection

This study was a retrospective analysis. Adult (age ≥ 18 years) patients treated at Princess Margaret Cancer Center between 2011 and 2021 were included if they had a confirmed diagnosis of localized or metastatic GEC and a baseline ESAS (defined as within 1 month before starting any treatment including any systemic anticancer therapy, radiation, or chemoradiation; Supplementary Fig. S1). The study was approved by the University Health Network Research Ethics Board (REB)—CAPCR ID 14-8075—and adhered to the data confidentiality and privacy policy of the International Credential Evaluation Service.

For each included patient, the clinical staging was determined using the American Joint Committee on Cancer (AJCC 6) staging manual, given the retrospective nature of this cohort.^21^ Esophageal cancer included adenocarcinoma of the distal esophagus and adenocarcinoma of esophagogastric junction types I and II (Siewert classification), while type III was defined as gastric cancer. Other histologies such as squamous cell carcinoma, adenosquamous carcinoma, neuroendocrine, undifferentiated carcinoma, and small-cell carcinoma were excluded from this analysis since they could have different prognoses.

Patient characteristics, including age at diagnosis, sex, ethnicity, and ECOG PS, were recorded. Tumor characteristics, including date of diagnosis, location, clinical staging, and HER2 status, were also noted. Patient-Reported Functional Status was added to supplement the ESAS survey.

### Outcomes

The primary objective of the study was to determine whether there is an association between the baseline SB and overall survival (OS) in patients diagnosed with gastroesophageal adenocarcinoma (GEA) from the time of diagnosis until their last follow-up or death. The study also aimed to assess the concordance between the PRFS measure and the physician-reported PS via ECOG assessment. Additionally, the study aimed to compare the effectiveness of 2 prognostic models, ESAS and physician-assessed ECOG, in predicting survival outcomes.

### Measures

#### Eastern Cooperative Oncology Group

The ECOG scale is commonly used to evaluate the PS of patients and is frequently incorporated in physician evaluations, eligibility requirements for clinical studies, and standard treatment recommendations. The ECOG scale has a range of 0-5, where 0 denotes fully functional and asymptomatic individuals, 4 represents those who are bedridden, and 5 means the patient is dead.^22^

#### Patient-reported functional Status

The PRFS is a tool for measuring PS that is completed by patients themselves. It is one component of the Patient-Generated Subjective Global Assessment (PG-SGA), which is a widely used and validated measure for assessing nutritional and functional status in patients with cancer. Originally developed in the 1990s, the PG-SGA includes both clinician and patient-completed components, with the activities/function domain (PRFS) based on the ECOG performance scale but expressed in simpler terms. Patients rate their own function on a scale of 0-4, with 0 indicating normal function and 4 indicating being bedridden (Supplementary Fig. S2).^19^

#### Edmonton Symptom Assessment System

The revised Edmonton Symptom Assessment System (ESAS-r) is an updated version of the ESAS that has been made available in multiple languages to aid in the evaluation of 10 prevalent symptoms. These symptoms include pain, tiredness, drowsiness, nausea, lack of appetite, shortness of breath, depression, anxiety, well-being, and constipation. By using a 0 to 10 scale, the ESAS-r allows individuals to express the severity of each symptom they are experiencing (Supplementary Fig. S3). The total SB can be calculated by adding up the scores for all 10 symptoms, resulting in a range of 0-100. Higher scores indicate more severe symptoms.^23^

### Statistical Analysis

Patient characteristics were summarized using descriptive statistics. Patients were grouped into 3 cohorts based on their ESAS scores as follows: high SB with ESAS score ≥ 26, moderate SB (11-25), or low SB (0-10). The SB categorization was done using tertiles, which are cut points that divide the range of a probability distribution into continuous intervals with equal probabilities.

OS was defined as the time from diagnosis to death from any cause. The Kaplan-Meier method was used for time-to-event analyses. The log-rank test was used to compare survival outcomes between SB groups. The Cox proportional hazards regression model was used to assess the association between patient characteristics and OS. Patients without documented evidence of an event were censored at the date of the last follow-up.

Univariable Cox proportional hazard models were conducted to calculate hazard ratios (HR) and respective 95% CI. To quantify the effect of each baseline ESAS composite score on OS in the presence of other covariates, multivariable Cox proportional hazard models were also performed. A statistical significance level of 5% (*P* < .05) was used.

The concordance index (c-index) was used to compare the prognostication impact of ESAS versus physician-ECOG PS. This was performed using the concordance statistic (C-statistic) derived from the Cox proportional hazards model, with OS as the outcome and ECOG PS as the predictor. To evaluate the agreement between PRFS and physician-reported ECOG PS ratings, weighted Kappa statistics were used, alongside a Bland-Altman plot (Supplementary Fig. S4) to display the level of agreement between the two.^24^ The difference between ESAS and physician-reported ECOG PS was statistically tested using a paired *t*-test.

## Results

### Demographic Characteristics

A total of 2000 patients were diagnosed with GEC between 2011 and 2021 at Princess Margaret Cancer Centre. Of these patients, 840 had at least 1 ESAS completed during their visits (Supplementary Fig. S1). ESAS is a long-standing tool used in Ontario before patient visits, but it is not mandatory. Patients have the choice to fill out the survey or not. Although many patients complete it, not all do so within the required 30-day period before treatment, as per eligibility criteria in this study. A total of 22 patients were excluded due to the presence of other neoplasia. A total of 211 patients had baseline ESAS and were included in this analysis.

Demographic and clinical characteristics of 211 patients with GEC are shown in Supplementary Table S1. The median age of the patients was 60.8 years (range: 29-82 years). The majority of patients were male (67.7%) and non-Asian (82%). Metastatic disease was present in 131 (62%) of patients at diagnosis, while 80 (38%) of patients had localized disease (stages I-III) at diagnosis. In terms of tumor location, gastric represented 47% of patients, gastroesophageal junction 39%, and esophageal 13%. Most physicians rated patients’ ECOG PS as 0 (32%) or 1 (58%). Among patients with known HER2 status, 74% were negative and 25% positive.

### Survival Analysis

**Figure 1.**
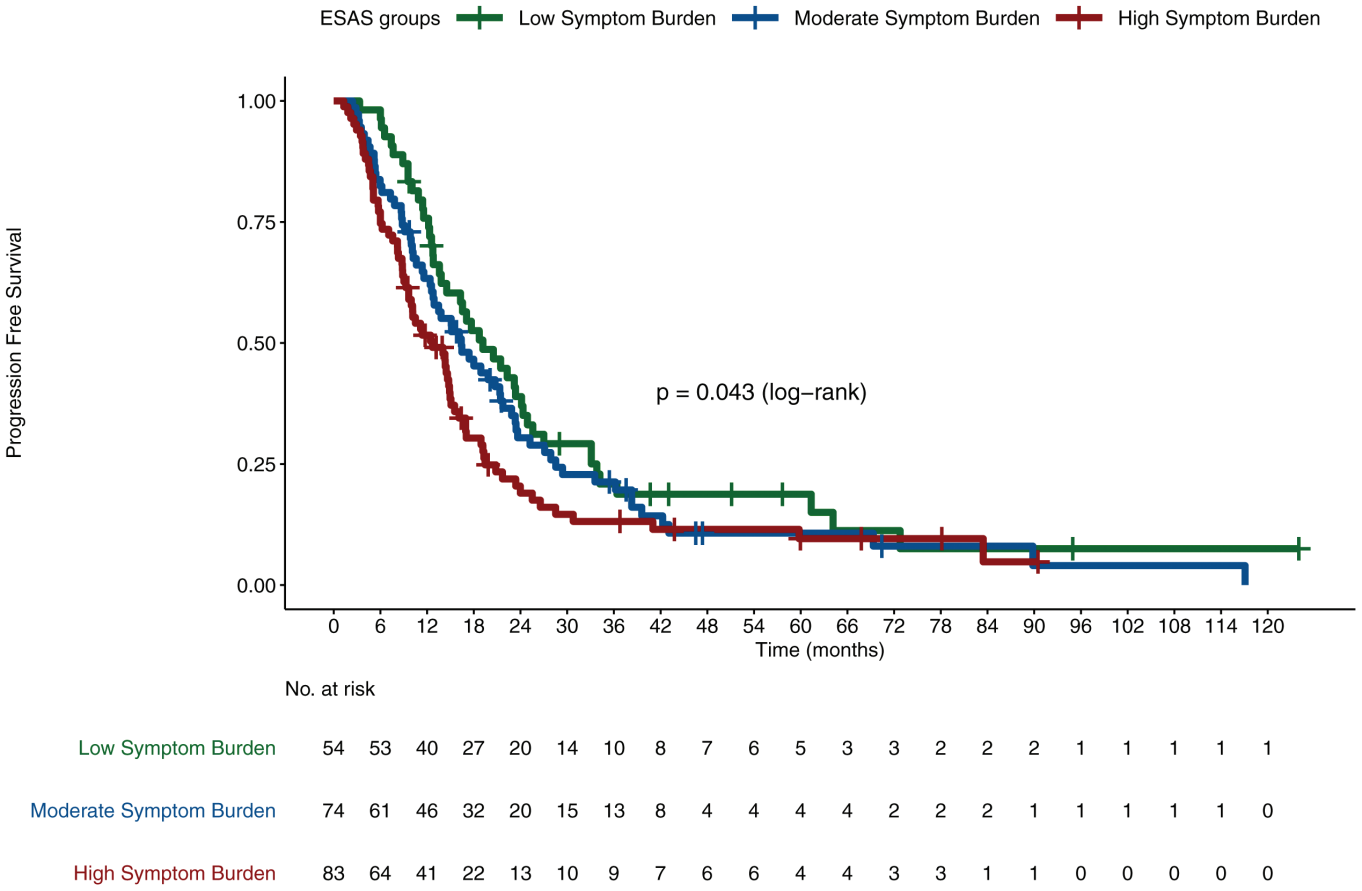
Overall survival of all patients stratified by ESAS SB groups.

**Figure 2.**
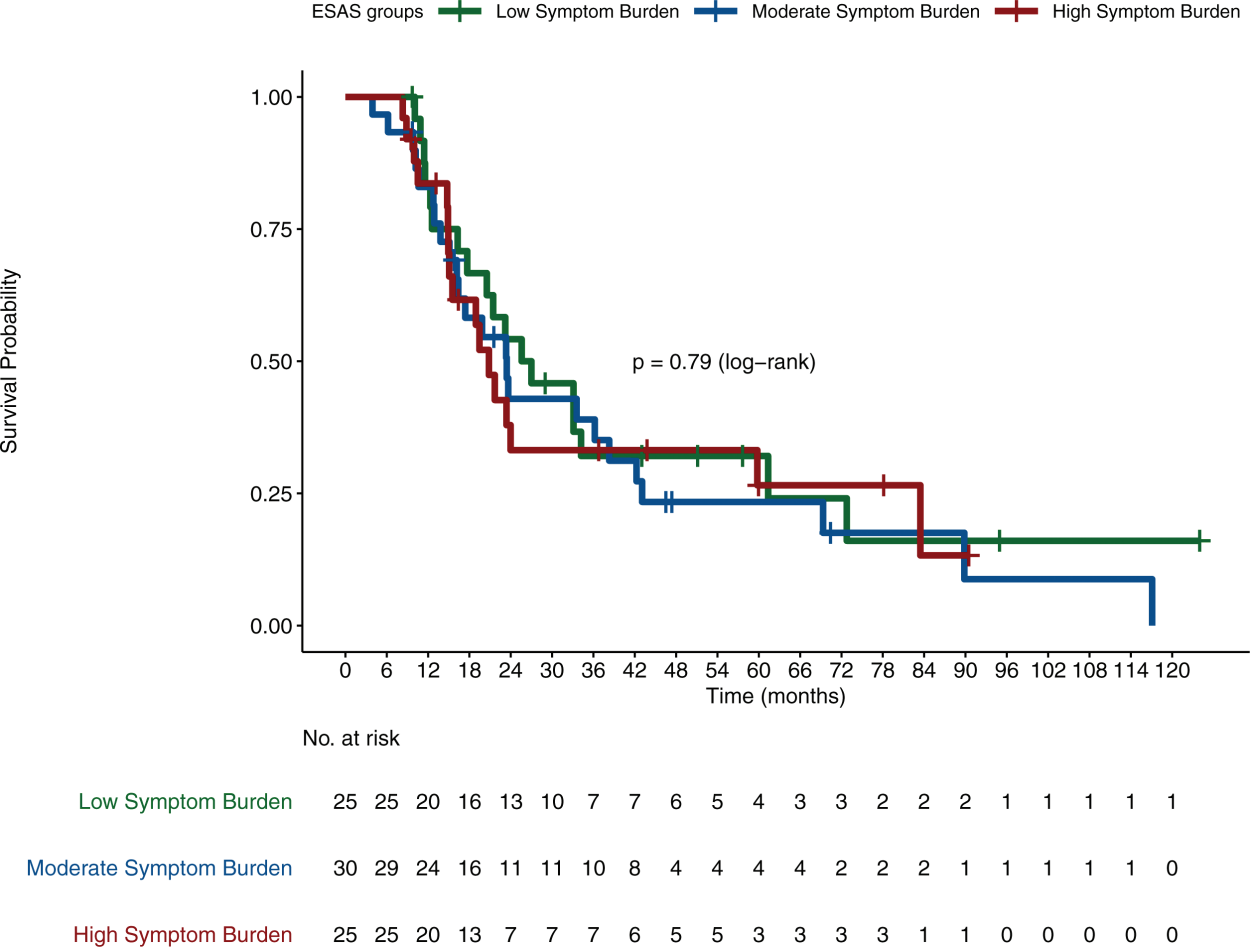
Correlation between SB and OS in localized patients.

**Figure 3.**
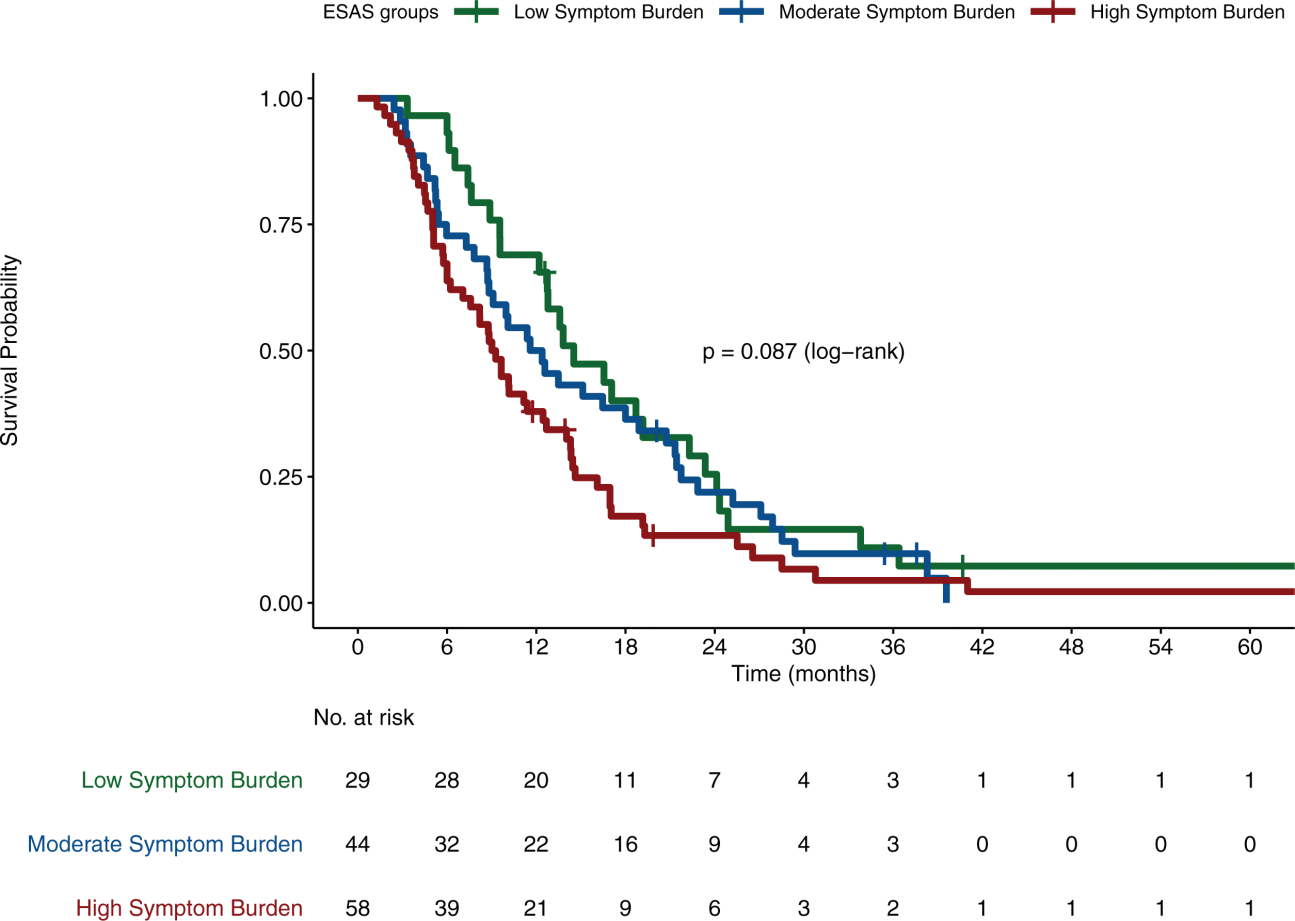
Correlation between SB and OS in metastatic patients.

Median OS in low, moderate, and high SB cohorts were 19.17, 16.39, and 12.68 months, respectively (*P* < .04; Fig. 1). Analyzed separately, worse ESAS levels did not correlate with worse OS for either localized patients (*P* = .79) or metastatic patients (*P* = .087; Figs. 2, 3, respectively).

We assessed median survival among patients rated with ECOG PS scores of 0, 1, and ≥2 by physicians. ECOG 1 yielded an HR of 1.22 (95% CI, 0.89-1.68), while ECOG ≥ 2 resulted in an HR of 2.56 (95% CI 1.49-4.40), exhibiting a significant global *P*-value of .02. Conversely, we conducted a similar analysis on patients self-rated with PRFS scores of 0, 1, and ≥2. PRFS 1 displayed an HR of 1.10 (95% CI, 0.73-1.65), whereas PRFS 2 showed an HR of 1.27 (95% CI, 0.81-1.99), yielding a non-significant global *P*-value of .57.

Univariate analysis revealed a significant association between OS and high SB cohort (HR: 1.60; 95% CI: 1.10-2.32, *P* = 0.01), ECOG PS ≥ 2 (HR = 2.56; 95% CI: 1.49-4.40, *P* < .0001), and the presence of metastatic disease (HR = 2.79; 95% CI, 2.00-3.88; *P* < .0001). The patients with HER2+ disease had a reduced risk of death (HR = 0.57; 95% CI, 0.39-0.84; *P* < .0001; Table 1).

**Table 1.** Univariable analysis.

	HR (95% CI)	*P*-value	Global *P*-value	*N*
Age		.66		211
≤60.8	Reference			105
>60.8	0.94 (0.70-1.26)			106
HER2 status		**.004**		168
Negative	Reference			125
Positive	0.57 (0.39-0.84)			43
Stage		**<.001**		211
Stage I-III	Reference			80
Stage IV	2.79 (2.00-3.88)			131
ECOG PS			**.004**	211
0	Reference			68
1	1.22 (0.89-1.69)	.22		123
≥2	2.56 (1.49-4.40)	**<.001**		20
ESAS groups			**.04**	211
1	Reference			54
2	1.23 (0.84-1.80)	.28		74
3	1.60 (1.10-2.32)	**.01**		83

The bold ones are significant (*P* <.05).

Abbreviations: ECOG PS, Eastern Cooperative Oncology Group; ESAS, Edmonton Symptom Assessment Scale; HER2, human epidermal growth factor receptor 2.

In the multivariable analysis (MVA), metastatic patients (stage IV) exhibited significantly poorer OS: 2.86 (2.04, 4.00) with a *P*-value of <.0001. Similarly, an ECOG PS ≥ 2 corresponded to an HR of 2.24 (1.29, 3.89) and a *P*-value of .004. Notably, when examining ESAS categorization, the global *P*-value stood at .08, lacking statistical significance. However, the ESAS category 3 (high SB) cohort separately showed an increased risk of death with an HR of 1.53 (1.05, 2.24) and *P*-value of .03 (Table 2).

**Table 2.** Multivariable analysis.

	HR (95% CI)	*P*-value	Global *P*-value	*N*
ESAS groups			.08	211
1	Reference			54
2	1.24 (0.84-1.81)	.28		74
3	1.53 (1.05-2.24)	**.03**		83
Stage		**<.001**		211
Stages I-III	Reference			80
Stage IV	2.86 (2.04-4.00)			131
Age	1.01 (1.00-1.02)	.18		211
ECOG physician			**.02**	211
0	Reference			68
1	1.28 (0.92-1.76)	.14		123
≥2	2.24 (1.29-3.89)	**.004**		20
Stage		**<.001**		211
Stages I-III	Reference			80
Stage IV	2.84 (2.02-3.98)			131
Age	1.01 (0.99-1.02)	.39		211

All bold ones are significant.

Abbreviations: ESAS, Edmonton Symptom Assessment Scale; HER2, human epidermal growth factor receptor 2; ECOG PS, Eastern Cooperative Oncology Group.

### Prognostic Comparison Between Physician-Reported ECOG PS and ESAS

The c-index for 2 models (ESAS vs physician-reported ECOG PS) were calculated and compared among 211 patients (Table 3). The C-statistic was similar between physician-reported ECOG PS (c-index 0.56) and ESAS (c-index 0.57) in their ability to predict OS in all patients. The results were similar in metastatic and localized patients. There were no statistical differences between the c-index of 2 models.

**Table 3.** Prognostication between ESAS and physician-reported ECOG PS (211 samples).

c-index (ECOG PS)	c-index (ESAS)	Diff	SD (diff)	Z test	*P*-value
All stages					
0.56	0.57	−0.00986	0.0248	−0.3977	.69
Stages I-III					
0.51	0.52	−0.00564	0.054	−0.1045	.91
Stage IV (metastatic)					
0.57	0.57	−0.00018	0.0276	−0.0066	.9772

### Performance Status Agreement and Prognostic Comparison Between Physicians and Patients

The agreement between physician- and patient-reported PS ratings was shown in Supplementary Table S2. In our study, there was a fair agreement between PRFS and physician-reported ECOG PS with a weighted Kappa of 0.28 (95% CI: 0.17-0.38).^25^ The total agreement between patient-reported PS and physician-reported PS is 50%. Thirty-six percent of the patients rated their score as worse and 14% of the patients rated their score as better than physicians did. On average, patients rated their scores (PRFS average = 2.18) as worse by 0.33 points (95% CI: 0.21-0.46, *P* < .0001) than physicians did (average ECOG PS = 1.85) as shown by the solid line in the Bland-Altman plot (Supplementary Fig. S4). When it comes to prognostication, the C-statistic was analyzed in 181 patient samples and showed a similarity between physician-reported ECOG PS (c-index 0.56) and PRFS (c-index 0.54) predicting death (Table 4).

**Table 4.** Prognostication between physician-reported ECOG PS versus PRFS (181 samples).

c-index (ECOG PS)	c-index (PRFS)	Diff	SD (diff)	*Z* test	*P*-value
0.56	0.54	−0.01984	0.0265	−0.7488	.4539

## Discussion

To our knowledge, this is the first study to investigate the association between SB and the risk of death in patients with gastroesophageal cancer (GEA). Understanding the prognosis of a patient with cancer is pivotal in the development of risk-based interventions that can enhance patient outcomes. Additionally, the knowledge of prognosis could stimulate more comprehensive conversations between healthcare providers and patients regarding the available treatment options and end-of-life care such as timely initiation of palliative care or hospice services.^23^ We evaluated 211 patients with GEC with metastatic and localized disease and examined the relationship between baseline PROs (measured by the ESAS survey) and survival rates. Our findings indicated that higher SB was linked to significantly shorter OS, highlighting the potential use of the ESAS-r survey as a dependable prognostic tool in patients with GEC.

While there was no significant statistical difference between metastatic and localized patients when analyzed in separate subgroups, there was a trend toward decreased OS and higher levels of ESAS especially in patients with metastatic disease. We believe that we would have more powerful results with a larger sample size of patients. In localized patients with GEC, we hypothesize that the impact of baseline SB on prognosis may be harder to establish when there are numerous other baselines competing risks that have been established to be associated with outcomes such as age, histological grade, T stage, tumor size, N stage, and lymph node involvement.^26^ Despite the challenges of proving this in the presence of other influential factors, it is possible that SB could still have a prognostic influence and also incorporate patients into their care. In patients with metastatic disease, baseline symptoms may play a greater role in determining OS. Studies have revealed that the prognosis is independent of HER2 expression^27^; however, HER2+ disease is a predictor of response to anti-HER2 agents in metastatic setting and patients live longer. Other studies suggested that the presence of lung or bone metastases is an independent risk factor for decreased survival.^28^ Additionally, while in the UVA, there was a significant association between worse OS and SB, the MVA showed that only patients with a high SB cohort had a significantly increased risk of death.

Despite the widespread use of PS scales in oncology practice to evaluate patients’ suitability for cancer trials, prognostication, response to treatment, self-care ability, and the requirement for home care services,^18,29^ the ECOG PS is a subjective scale.^30^ Additionally, ECOG PS is a limited unidimensional tool that disregards crucial domains such as multimorbidity, aging, frailty, and psychological health.^31^ Moreover, as the ECOG PS is mainly assessed by physicians, it is subject to bias and does not consider patients’ PS self-perceptions. The PRFS was added to supplement the ESAS survey and is a tool which constitutes the function aspect of the Patient-Generated Subjective Global Assessment (PG-SGA) measure.^9^ This indicates that the ESAS survey provides not only patient-centered well-being but also reflections on patients’ physical functioning.^10^ Our study showed that there was an interobserver variability which was considered a fair agreement between PRFS and ECOG PS based on weighted kappa. Total agreement in patient and physician reports of PS was found for 50% of assessments; this result is consistent but slightly higher than the literature,^18,20^ in which agreement ranged from 37% to 42%. In our study, as in others, patients tended to rate their PS as worse than that rated by physicians. The disparity between clinicians’ and patients’ perceptions in assessing PS has brought up important questions. As PS plays a vital role in medical and patient decision-making, including the patient’s self-assessment in the decision-making process enables them to actively participate in informed decisions about their treatment. It is noteworthy that, despite a similar c-index between PRFS and physician-reported ECOG PS, the predictive power for survival was only significant in physician-reported ECOG PS (Supplementary Figs. S5, S6). In contrast, within the PRFS group, the HR exhibited a numerical tendency but did not reach statistical significance. In a larger population study, the literature has demonstrated a strong correlation between both PRFS and physician-ECOG scores and OS.^18^

In our study, there were no discernible differences between the ESAS and physician-reported ECOG PS models in terms of prognostication. However, ESAS adds the ability to quantify multiple symptoms efficiently and systematically and has revolutionized symptom assessment in both clinical practice and research, leading to its widespread adoption in the past 30 years.^32^ Additionally, the ESAS is increasingly used to trigger specific clinical actions, such as referring patients to palliative care teams.^33^ Even so, despite its benefits, most PS and prognostic measures are still clinicians-centered. ESAS is PROM that enables the capture of the patient’s perspective and is a valuable tool for patients with GEC in the present study, with the potential to be integrated into their care.

The strength of our study is the utilization of real-world data to support the integration of PROs into routine clinical care. However, it has certain limitations due to its retrospective design, which makes it difficult to account for potential confounding factors and inherent selection bias that may have influenced the result such as the lack of available information on comorbid conditions and other clinical variables for all patients. Although the difference was not statistically significant when comparing metastatic and localized patients separately, there was a numerical trend toward poorer OS and worse ESAS levels, particularly in patients with metastatic disease. The relatively small sample size of the study may have contributed to these findings. Additionally, the cutoff points for categorizing patients into high, moderate, and low SB groups were based on tertiles, as limited data defines these categories based on ESAS total score. To better comprehend the independent prognostic value of PROs, future prospective studies that consider all major prognostic factors are necessary.

## Conclusion

This study suggests that a higher baseline SB, as measured by the total ESAS score, is associated with reduced survival in gastroesophageal patients. These results underscore the importance of patient-reported outcome measures in predicting prognosis. Clinicians and patients commonly disagree on how to assess a patient’s PS. While the ESAS and physician-reported ECOG PS models showed comparable prognostic value, the use of ESAS has the potential to advance patient-centered cancer care.

## Supplementary Material

Supplementary material is available at *The Oncologist* online.

oyae010_suppl_Supplementary_Figures

oyae010_suppl_Supplementary_Tables

## Data Availability

The data underlying this article will be shared on reasonable request to the corresponding author.
