# Supplementary material for: Integrating Patient-Reported Outcomes Into Prognostication in Gastroesophageal Cancer: Results of a Population-Based Retrospective Cohort Analysis
Source: Oncologist. 2024 Mar 2;29(4):316–23. doi: 10.1093/oncolo/oyae010 (PMC10994401; doi:10.1093/oncolo/oyae010)

**Supplemental Figure 1**. *Flowchart of Included Patients*


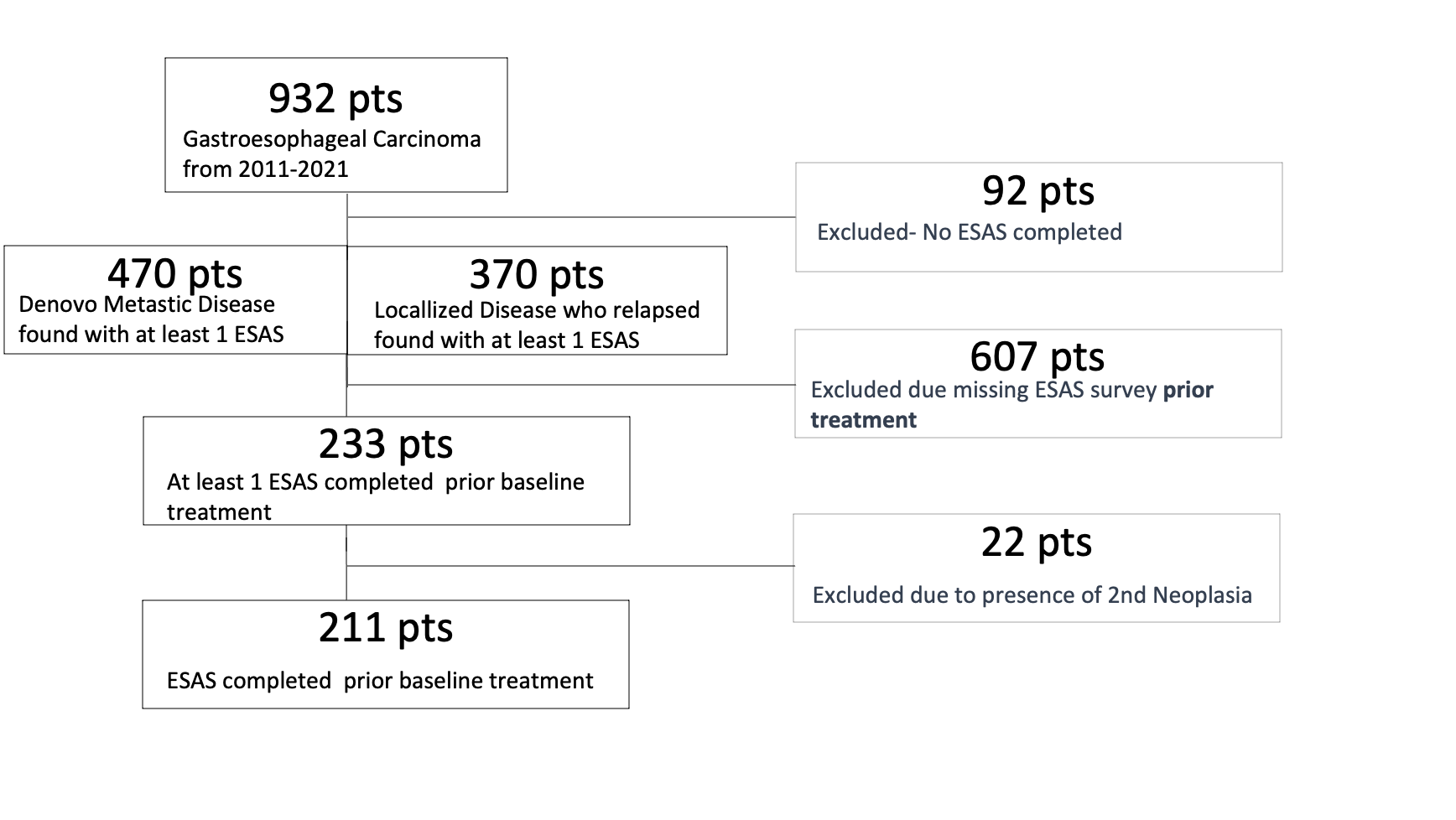


*Acronyms: ESAS, Edmonton Symptom Assessment Scale.*

**Supplemental Figure 2**. *Patient-Reported Functional Status (PRFS) questionnaire.*


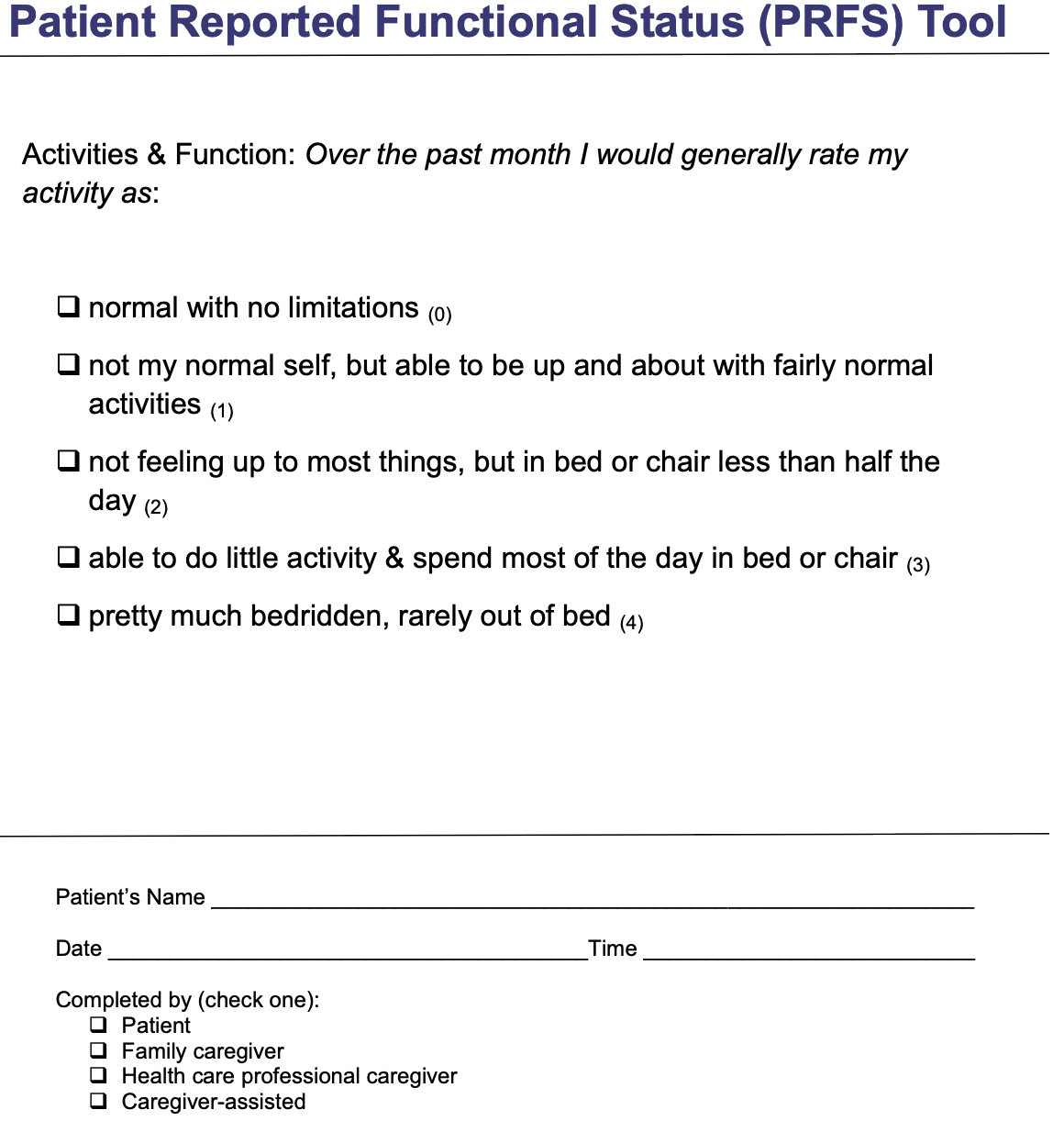


**Supplemental Figure 3.**  *Edmonton Symptom Assessment System (ESAS) questionnaire*


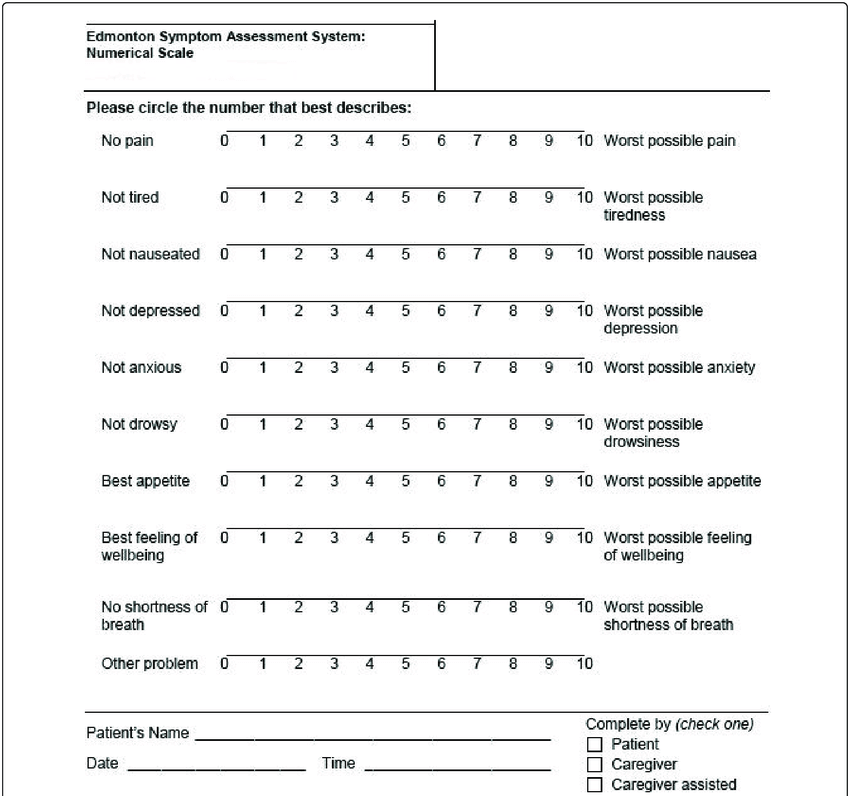


**Supplemental Figure 4.** Difference between Physician and Patient’s reported performance status


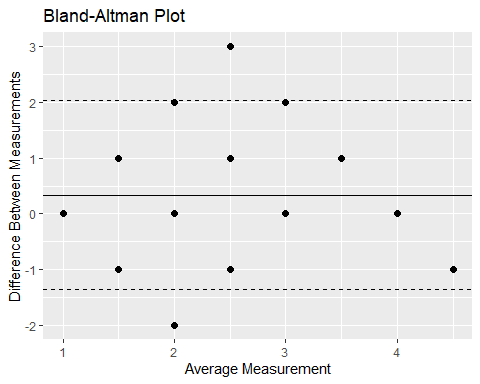


*Bland-Altman plot of difference in performance status vs average of patient and physician-reported performance status.*

**Supplemental Figure 5.** *Correlation between ECOG performance status rated by the physicians and Overall Survival*

*
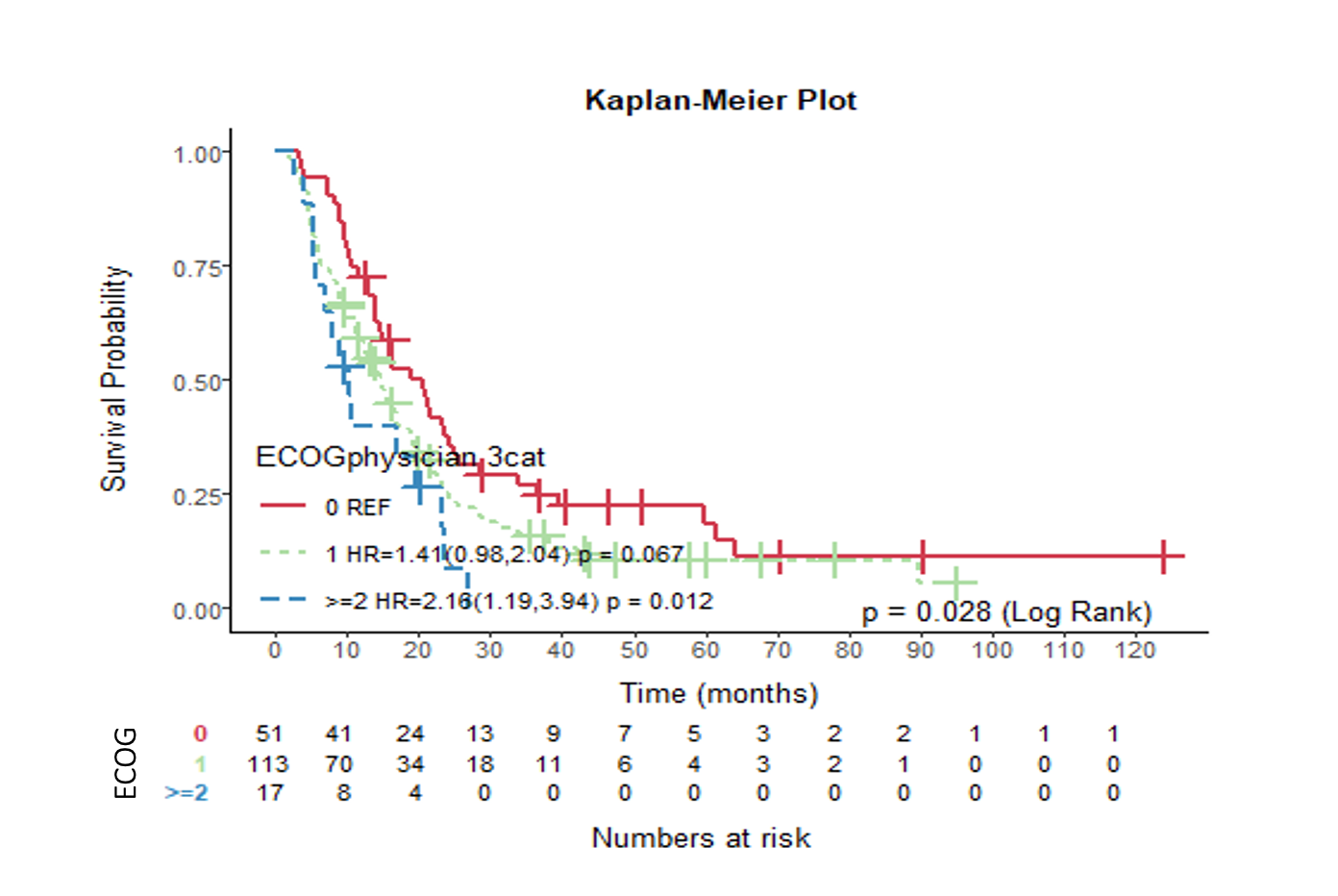
*

***Supplemental Figure 6.*** *Correlation between patient-reported functional status Overall Survival*


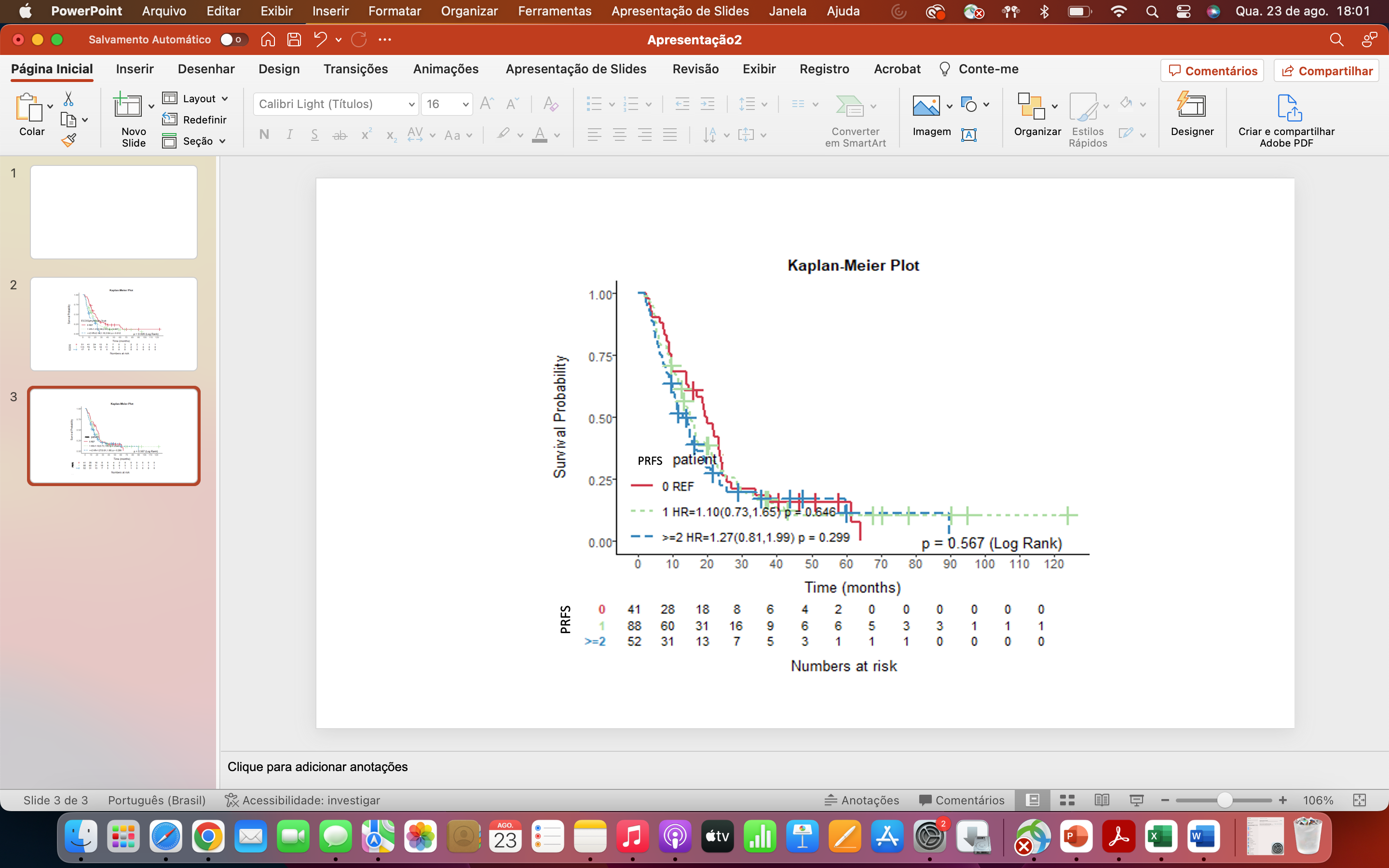

Supplement: oyae010_suppl_Supplementary_Figures [file oyae010_suppl_supplementary_figures.docx]
