# Supplementary material for: Integrating Patient-Reported Outcomes Into Prognostication in Gastroesophageal Cancer: Results of a Population-Based Retrospective Cohort Analysis
Source: Oncologist. 2024 Mar 2;29(4):316–23. doi: 10.1093/oncolo/oyae010 (PMC10994401; doi:10.1093/oncolo/oyae010)
Supplement: oyae010_suppl_Supplementary_Tables [file oyae010_suppl_supplementary_tables.docx]

**Supplemental Table 1.** *Characteristic of Included Patients*

|  | Total (n=211) |
| --- | --- |
| **Age category** |  |
| <= 60 | 105 (50) |
| > 60 | 106 (50) |
| **Race** |  |
| Asian | 39 (18) |
| Non-Asian | 172 (82) |
| **HER2** |  |
| Negative | 125 (74) |
| Positive | 43 (26) |
| Missing | 43 |
| **Localization** |  |
| Esophagus | 28 (13) |
| Gastric | 100 (47) |
| GEJ | 83 (39) |
| **Stage** |  |
| Stage I-III | 80 (38) |
| Stage IV (metastatic) | 131 (62) |
| **ECOG PS baseline** |  |
| 0 | 68 (32) |
| 1 | 123 (58) |
| 2 | 16 (8) |
| 3 | 3 (1) |
| 4 | 1 (0) |
| **ESAS groups** |  |
| 1 | 54 (26) |
| 2 | 74 (35) |
| 3 | 83 (39) |

*Acronyms: ESAS, Edmonton Symptom Assessment Scale; ECOG PS, Eastern Cooperative Oncology Group; HER2, human epidermal growth factor receptor 2*

**Supplemental Table 2**: *Agreement between Performance status by patients (PRFS) and by physicians (ECOG PS)*

|  | **ECOG PS** | | | | |  |
| --- | --- | --- | --- | --- | --- | --- |
| **PRFS** | 0 | 1 | 2 | 3 | 4 | Total |
| 0 | 24 | 17 | 1 | 0 | 0 | 42 |
| 1 | 20 | 63 | 6 | 0 | 0 | 89 |
| 2 | 6 | 23 | 2 | 0 | 0 | 31 |
| 3 | 1 | 12 | 5 | 3 | 1 | 22 |
| 4 | 0 | 0 | 0 | 0 | 0 | 0 |
| Total | 51 | 115 | 14 | 3 | 1 | 184 |

The weighted Kappa is 0.27 (95% CI:0.17- 0.38), which is considered as fair agreement [6]. The total agreement between patients and physicians is 50%. 36% of the patients rated their score as worse and 14% of the patients rated their score as better than physicians did. On average, patients rated their scores as worse by 0.33 points (95% CI: 0.2-0.45, P<0.0001) than physicians did (ECOG-physician=1.84)

*Acronyms: PRFS = patient-reported functional status; ECOG PS = Eastern Cooperative Oncology Group (physician-reported functional status).Note: Shaded numbers indicate agreement between physician and patient scores*
